# The effects of images posted to social media by orthodontists on public perception of professional credibility and willingness to become a client

**DOI:** 10.1186/s40510-021-00353-9

**Published:** 2021-03-08

**Authors:** Thiago Martins Meira, Jeany Prestes, Gil Guilherme Gasparello, Oscar Mario Antelo, Matheus Melo Pithon, Orlando Motohiro Tanaka

**Affiliations:** 1 School of Life Sciences, Pontifícia Universidade Católica do Paraná, Curitiba, Brazil; 2 Bahia State University (UNEB), Guanambi, Brazil; 3 Department of Orthodontics, Universidad Católica Boliviana San Pablo, Santa Cruz de La Sierra, Bolivia; 4 Department of Orthodontics, Southwest Bahia State University (UESB), Jequié, Brazil; 5 Graduate Dentistry Program in Orthodontics, PUCPR, R. Imaculada Conceição, 1155, Curitiba, PR 80215-901 Brazil

**Keywords:** Orthodontics, Social media, Health services marketing

## Abstract

**Background:**

Many patients choose health professionals using the Internet, whether through websites or social media. In orthodontics, an orthodontist’s relationship with active and potential patients can be affected by social media interactions, both as a marketing tool and as a tool for providing educational information. The purpose of the present study was to analyze the public perception of professional credibility and willingness to become a client, based on images posted by orthodontists on Instagram.

**Method:**

This was a cross-sectional study performed using a digital self-administered questionnaire based on images from public Instagram profiles of orthodontists found using certain hashtags. The themes of the posts were analyzed through a qualitative analysis, and the results were expressed as categories. After analyzing 2445 images, 12 thematic categories emerged. A total of 446 individuals (225 laypeople, 66 dental students, and 155 dentists) evaluated the images in regard to the perception of professional credibility and willingness to become a client. One-way ANOVA and chi-square tests were applied, considering a 5% significance level.

**Results:**

It was found that more than 95% of the participants used social media, primarily Instagram, WhatsApp, and Facebook, and the social network most used to research health services was Instagram. Statistically significant differences were found in the mean value of perceived professional credibility between the groups (*p* < 0.05) for the following categories: “dental traction,” “mini-implant mechanics,” “before and after treatment,” “aesthetic brackets,” “metallic brackets,” and “clear aligners.” The categories “being a teacher” and “before and after treatment” had a higher impact on the participants’ perception of credibility and willingness to become a client, unlike the “social relationship” and “family relationship” categories.

**Conclusions:**

Some of the themes found in the orthodontists’ social media posts were found to influence the perceptions around professional credibility and willingness to become a client, although there were differences among the participants in the present study.

## Background

Prior to the popularization of the Internet, orthodontists attracted potential patients through social means or referrals from other professionals. In the digital age, however, many patients find healthcare professionals using the Internet, either through websites or social media [1]. Recently, most orthodontists, and patients or their parents, use social media, which can be an effective marketing and communication tool for orthodontic practices [2]. However, we wanted to assess the impact that social media has had on the effort of orthodontists to portray their professional credibility to potential patients. Is it possible that, through posting on social media, there is a greater possibility of these professionals attracting new patients [3]?

In the dental field, social media postings seem to be useful for developing and maintaining the loyalty of current patients and for helping potential patients to learn about professionals’ clinical practices [4]. The social media outlet most frequently used by both orthodontists and their patients is Facebook, which is thought to be positively related to acquiring new patients [2]. Additionally, social media can also function as an educational tool for orthodontic patients [5].

Social media has been found to be very important in regard to orthodontists’ relationships with active and potential patients, both as a marketing tool and as a tool for providing educational information [2, 4, 5]. Recently, Instagram has become a dominant channel for business marketing targeting young adults, and its use in the aesthetic medical field continues to increase [6]. Instagram is one of the social media outlets most used by teenagers, a population that has a preference for receiving oral health instructions through social media [7].

Instagram, similar to Facebook and Twitter, uses hashtags that permit users to search for content of interest. When a specific hashtag is used, Instagram automatically lists the total number of posts using that hashtag, as well as the “top” or most popular posts, followed by the remaining posts, using this hashtag, which are then sorted in chronological order [6].

Due to the increased influence of social media on healthcare in recent years, researchers have begun to investigate its impact on professional credibility and the professional-patient relationship. They have pointed out that the public’s perceptions of professionalism and credibility are essential for the development of guidelines regarding professionalism in digital media, and to encourage best-use practices for social media [3].

The digital age of dentistry is currently a reality, and sooner or later, all orthodontists will make direct or indirect use of digital technologies, including social media [8]. However, it is of fundamental importance to know if the content posted by professionals in this field generates the expected credibility regarding the perceptions of their potential patients, or even if there is any influence on the possibility of these becoming their future clients. To the best of our knowledge, this is a precursor study in orthodontics.

Therefore, the objective of the present study was to analyze the public perception of professional credibility and willingness to become a client based on images commonly posted on Instagram by orthodontists.

## Methods

This cross-sectional study was approved by the university’s ethics committee. The target study population was individuals over 18 years of age and of any race, education, or social level. Laypeople were included, as were dental students and dental professionals from Brazil. A sample calculation was performed to define the number of individuals targeted to participate in the research. A sample number of *n* = 380 was obtained, considering an infinite population, a margin of error of 5%, a confidence level of 95%, and a prevalence of 50%.

A self-administered questionnaire developed by the authors according to the study of Weijs et al. [3] using the Qualtrics digital platform (Salt Lake, UT, USA) was used for data collection. Participants could access the questionnaire digitally using a computer, smartphone, or tablet. The variables were the perception of credibility and the motivation to consult with a professional based on categorized images commonly posted on social media by orthodontists.

Instagram was searched for the public profiles of orthodontists. All profiles included for analysis were freely accessible to the general public on the Internet, with the images available for viewing. In order to find a greater number of professional profiles, orthodontic-related terms were searched in English, Spanish, and Portuguese in the form of the hashtags #ORTHODONTICS, #ORTODONCIA, and #ORTODONTIA. Public profiles of orthodontists who had more than 15,000 followers were selected for the present study, and these orthodontists were considered digital influencers.

Posts were included in our analysis if they were relevant to orthodontics (i.e., intra- or extra-oral clinical images of treatments, photos before and after treatment, explanatory diagrams of techniques or treatments, and posts of interpersonal relationships between orthodontists and patients). Duplicate posts, advertisements for companies or materials, images of landscapes, and posts about hobbies were excluded from the present study as well as posts in disagreement with legislation and code of ethics [9, 10].

The themes were ascertained using image analysis, and the results were expressed as categories [11]. Thus, we sought to understand the essence of what was being communicated through the posted images. After identifying and viewing the professional profiles of orthodontists on Instagram, the relevant images contained in the posts were analyzed. The categories to be evaluated were ascertained based on the repeated pattern of themes found in the images. The most relevant images for each category were saved in a research database.

Image selection and analysis were performed by two researchers (a dentistry student and an orthodontist). In the event that there was any disagreement regarding the categorization of images, a third researcher aided in reaching a consensus. Different professional profiles were analyzed until no new categories emerged (saturation) [12].

A total of 2445 images were analyzed, from the profiles of 10 national and international orthodontists. From the image analysis, the following 12 categories emerged:

- Intermaxillary elastics (intraoral images of the lateral or frontal view of treatments using fixed orthodontic appliances and intermaxillary elastics in different applications)
- Metallic brackets (extra-buccal images of a close-up smile illustrating the use of fixed-metallic orthodontic braces without focusing on mechanical aspects);
- Aesthetic brackets (intraoral treatment images of a fixed aesthetic device without focusing on mechanical aspects)
- Clear aligners (extra-buccal images of a close-up smile illustrating the use of transparent aligners without focusing on mechanical aspects)
- Social relationships (images of the orthodontist socializing with other people in informal settings, such as restaurants, parties, and bars)
- Relationships with the patient (images of the orthodontist in a lab coat with patients in his office)
- Family relationships (images of the orthodontist with his family in an environment outside the office)
- Mini-implant mechanics (intraoral images illustrating orthodontic treatment with a fixed appliance in which a mini-implant is being used as an anchorage)
- Participation in scientific events (images of the orthodontist at scientific events wearing formal attire with credentials)
- Being a teacher (images of the orthodontist teaching)
- Before and after treatment (intraoral images of orthodontic treatments using fixed appliances illustrating the initial appearance of malocclusions and their appearance after orthodontic correction)
- Dental traction (occlusal images illustrating orthodontic treatments using fixed braces and teeth being pulled into the occlusion line with the use of rubber bands).

A representative image was chosen for each thematic category, which was accompanied by two questions on the questionnaire: (1) For the social network of an orthodontist, does this type of post convey professional credibility, that is, does it appear to be a competent and reliable professional? and (2) Would this type of post motivate you to consult with this professional to put an orthodontic appliance in case it was necessary? For the first question, the answer was selected on a visual analog scale ranging from 0 to 10, where 0 represented no credibility and 10 represented total credibility. For the second question, there were three answer options (yes, no, and I do not know).

### Reliability

For validation and reliability testing, the questionnaire was administered twice during the preliminary stage of data collection, with an interval of 20 days between the 2 administrations. For the first (test) and second (retest) administrations of the questionnaire, 30 subjects were included, who were not included in the final sample.

### Data collection

For data collection, the questionnaire link was distributed via social media (Facebook, Instagram, LinkedIn, and WhatsApp) and email. The email addresses were listed from the annals of the 35th Annual Meeting of the Brazilian Division of the International Association for Dental Research (SBPqO-IADR) which included email addresses of professionals throughout all the country. The researchers forwarded the link to the questionnaire to their contacts and groups through social media.

### Data analysis

The collected data were tabulated in an electronic database using Microsoft Excel (Microsoft, Inc., Redmond, WA, USA). Statistical analysis was performed using SPSS version 25 (IBM, Armonk, USA). For reliability, the intraclass correlation and kappa coefficients were used, and Cronbach’s alpha was used to assess internal consistency. Descriptive statistics were performed by calculating the total count and percentage of qualitative variables, and the mean and standard deviation of the numerical variables. One-way ANOVA and chi-square tests were performed to compare the dependent variables (credibility and willingness to become a client) between groups. A significance level of 5% (*p* < 0.05) was adopted.

## Results

The questionnaire showed satisfactory internal consistency for all image categories evaluated (Cronbach’s alpha > 0.70). Satisfactory agreement rates were also found between the test and retest results.

A total of 446 individuals participated in the research, of whom 225 were laypeople, 66 were dental students, and 155 were dentists (31 clinicians, 45 orthodontists, and 79 specialists in other areas). Of the participants, 61.8% (*n* = 276) were women and 38.2% (*n* = 170) were men. The average age was 31.1 years. A significant difference (*p* < 0.05) in the average age between the groups was observed. Students (23.4 ± 6.2 years) and clinicians (27.2 ± 7.7 years) were significantly younger than laypeople (32.2 ± 12.5 years), orthodontists (36.7 ± 7.7 years), and other specialists (32.6 ± 8.2 years).

**Fig. 1:**
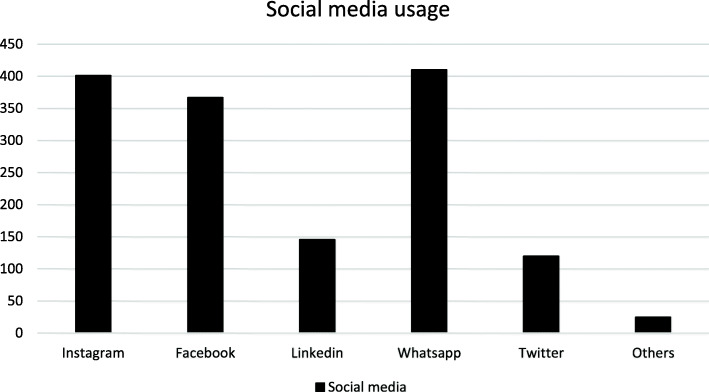
Distribution about social media usage of all participants

The distribution of the research population in relation to the usage of social media is described in Fig. 1. A total of 50.4% (*n* = 225) of the participants used social media to research health services, primarily Instagram (63.1%, *n* = 142) and Facebook (19.1%, *n* = 43). Dental students (67.7%, *n* = 44) and clinicians (67.9%, *n* = 19) used social media significantly more (*p* < 0.05) than laypeople (47.2%, *n* = 103), orthodontists (48.8%, *n* = 21), and other specialists (47.28%, *n* = 103) to research health services.

**Fig. 2:**
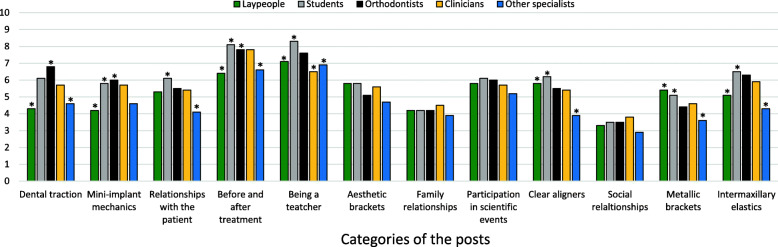
Distribution of the mean score for perception of credibility between groups, according to the different categories of the posts. Asterisk indicates statistical significance

There was a significant difference in the perception of credibility among participants, the results of which are shown in Table 1 and Fig. 2. Laypeople and specialists in other areas assigned lower values to all variables except for “aesthetic brackets,” “clear aligners,” and “metallic brackets.” In contrast, orthodontists and students assigned higher values to all variables, except for the three last variables mentioned. Higher scores were observed for the variables “being a teacher” and “before and after treatment,” and there was a significant difference between groups. Orthodontists and students attributed significantly higher values than laypeople and other specialists. The variables “social relationships” and “family relationships” had lower scores for all groups. A greater difference (*p* < 0.05) was observed in the perception of credibility between orthodontists and laypeople in the categories related to techniques such as “dental traction” and “mini-implant mechanics.”

**Table 1:** Results of the mean score for perception of credibility between groups, according to the different categories of the posts

Categories	Laypeople	Students	Orthodontists	Clinicians	Specialists in other areas	*p* value
	Mean (SD)	Mean (SD)	Mean (SD)	Mean (SD)	Mean (SD)	
Dental traction	4.3 (3.1)a	6.1 (2.9)b	6.8 (2.9)b	5.7 (3.1)ab	4.6 (2.9)a	0.000*
Mini-implant mechanics	4.2 (3.1)a	5.8 (2.9)b	6.0 (3.4)b	5.7 (2.7)ab	4.6 (3.1)ab	0.000*
Relationships with the patient	5.3 (3.2)	6.1 (2.7)a	5.5 (3.6)	5.4 (2.9)	4.1 (3.3)b	0.001*
Before and after treatment	6.4 (3.3)a	8.1 (2.2)b	7.8 (2.4)bc	7.8 (2.7)abc	6.6 (2.9)ac	0.000*
Being a teacher	7.1 (2.6)a	8.3 (1.9)b	7.6 (2.7)ab	6.5 (3.1)a	6.9 (2.9)a	0.004*
Aesthetic brackets	5.8 (3.1)	5.8 (3.1)	5.1 (2.9)	5.6 (3.1)	4.7 (3.1)	0.063
Family relationships	4.2 (3.1)	4.2 (3.4)	4.2 (3.3)	4.5 (2.8)	3.9 (3.3)	0.946
Participation in scientific events	5.8 (3.1)	6.1 (2.3)	6.0 (3.1)	5.7 (3.3)	5.2 (3.3)	0.545
Clear aligners	5.8 (3.2)a	6.2 (3.0)a	5.5 (3.2)a	5.4 (3.0)ab	3.9 (3.1)b	0.000*
Social relationships	3.3 (2.8)	3.5 (2.8)	3.5 (2.6)	3.8 (3.0)	2.9 (2.7)	0.521
Metallic brackets	5.4 (3.1)a	5.1 (2.9)a	4.4 (2.8)ab	4.6 (3.1)ab	3.6 (2.8)b	0.000*
Intermaxillary elastics	5.1 (3.2)ac	6.5 (2.8)bc	6.3 (2.9)ac	5.9 (3.3)abc	4.3 (3.1)a	0.000*

*Statistical significance; *SD* standard deviation; different letters mean statistical significance

**Fig. 3:**
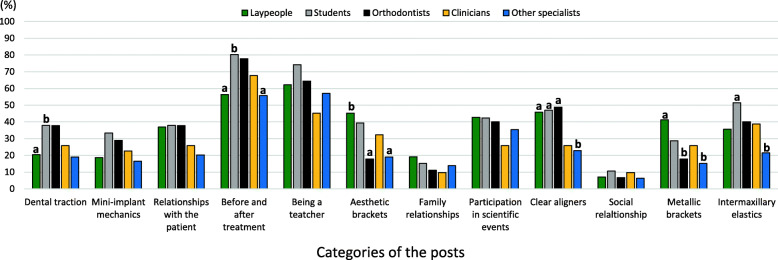
Distribution of the variable “willingness to become a client” between groups, according to the different categories of the posts. Different letters mean statistical significance

The results regarding the willingness to become a client based on the categories of the posts are shown in Fig. 3. More favorable results were associated with the variables “being a teacher” and “before and after treatment.” Orthodontists and dental students had higher scores. Posts related to “clear aligners” had higher results for orthodontists, students, and laypeople. In contrast, clinicians and other specialists had significantly more preference for posts such as “aesthetic brackets” and “metallic brackets” than orthodontists and other specialists (*p* < 0.05). Inversely, students and orthodontists had more preference for posts such as “dental traction,” “before and after treatment,” and “intermaxillary elastics” than laypeople, clinicians, and other specialists.

## Discussion

In recent years, social media, especially Instagram, has become an important tool for patients and professionals in the field of healthcare [6, 13]. The results of the present study indicated that the type of content published by orthodontists on social media can influence various groups of people differently, specifically in regard to professional credibility and the likelihood of selecting a professional for treatment.

Professional credibility is a broad concept formed by three main elements: caring, competence, and trustworthiness [14]. In the context of communication through social media, characteristics indicating credibility can strengthen the appearance of professionalism and thus increase patients’ trust in professionals in the health field [15]. The present study used images commonly posted on the publicly visible profiles of orthodontists. Although images alone are not enough to attest to the credibility of a health professional, Instagram is a predominantly visual social media platform, as the inclusion of text is optional. Therefore, the posted images transmit a significant amount of information to users [16]. Evaluating the public’s perception of professionalism and credibility is essential for the development of guidelines regarding professionalism in digital media and for encouraging best-use practices for social media [3].

We found similarity in the profiles of social media use between the participants included in the present study. More than 95% of the participants used social networks, especially Instagram and Facebook, and almost half of them used these social networks to seek health services. We found Instagram to be the social network most used to seek health services. In recent years, Instagram has gained great popularity worldwide, and many professionals and companies have begun to use this social network for commercial reasons [16]. According to Alalawi et al. [17], from the patient’s perception, the presence of the dentist on social media and an appropriate interaction with them is an important way to communicate with and reach new patients.

There were statistically significant differences in the perception of credibility between the groups. In categories related to specific mechanical factors of orthodontic treatments, such as “mechanics with mini-implants,” “dental traction,” and “intermaxillary elastics,” laypeople and specialists in other areas assigned significantly lower values when compared to orthodontists and dental students. On the other hand, categories related to general orthodontic treatments, such as “aesthetic brackets,” “metallic brackets,” and “clear aligners,” were more highly valued by laypeople when compared to other participants. Regarding these variables, clinicians and orthodontists had a similar perception.

These results can be explained by the fact that individuals who have experience with the dental field have a better understanding of the technical aspects of treatments and can better relate them to the image of a professional with probable skills and competence. This did not happen with laypeople, who assigned a lower correlation between these images and credibility, likely because they are unfamiliar with the technical specifics of the field. However, experts in other areas also did not value these types of technical posts and gave lower scores for credibility for most posts, showing that they have a different perception compared to their peers.

The categories most related to the perception of greater credibility for all groups were “being a teacher” and “before and after treatment.” These results show that this type of post can be positive for an orthodontist’s professional image, although laypeople, clinicians, and specialists in other areas assigned significantly lower scores than dental students and orthodontists to these variables. Other studies have found similar results that content about a dentist’s qualifications and before and after images were considered very important to patients when looking at a dental practice’s social media account [17]. In addition, posts related to “participation in scientific events” also had a better perception of credibility with no statistical difference between the groups.

Through social media, dental surgeons can advertise their professional trajectories, fields of work, and treatment procedures and techniques, focusing on information that educates the general population in a manner that does not typify the modification, unfair competition, or devaluation of the dental profession [9]. Ethical aspects are very important when discussing e-professionalism [10, 18]. The most discussed themes regarding the ethical application of Internet and communication technologies in healthcare are patient privacy, confidentiality and anonymity, data security, and informed consent [19]. Our findings showed that some post themes are more efficient in providing the credibility of the orthodontists on Instagram. However, the principles of ethics and code of professional conduct of each country must first be respected.

The findings of the present study showed that images related to social and family relationships were associated with lower scores regarding the perception of professional credibility for all groups. These results indicate that personal images, possibly because they are not related to the professional context, contribute little towards the professional image of orthodontists on Instagram. The General Dental Council guidelines on the use of social media also suggest that professionals must ensure that their behavior maintains their confidence and that of the dental profession [18].

Regarding the participants’ willingness to become an orthodontist’s patient based on the category of the posted images, these were in line with the categories that achieved the highest values for professional credibility for all groups (“being a teacher” and “before and after treatment”). However, orthodontists and dental students had more preference for these posts than the other groups. In addition, significant differences were observed between the opinions of laypeople and those of orthodontists and specialists in other areas of dentistry regarding categories that did not involve technical aspects, such as “metallic brackets” and “aesthetic brackets.” These findings showed that laypeople are enticed by images of the face that represent general orthodontic treatments, not the mechanical aspects of the treatment.

Weijs et al. [3] analyzed the perception of credibility based on comments by health professionals on Facebook and observed that credibility had a major influence on the participants’ desire to become a client of these health professionals. They also concluded that positive comments related to a professional’s daily work were reflected as a perception of greater credibility [3]. In another study, aspects of health professionals’ social media posts that diminish professionalism were identified, such as personal images with alcoholic beverages or those showing health professionals in bathing suits [20]. In the present study, the images that portrayed social and family relationships were found to be those that resulted in lower perceived professional credibility for all groups and were also less likely to motivate participants to select those professionals for treatment.

This study had some limitations. Credibility is a complex outcome and is difficult to measure from images only. Thus, our approach consisted of the verification of the participant’s perception about this outcome, and thus, the results had to be interpreted with caution. Another limitation was the restriction in the distribution of the questionnaire to only one country, despite which the results are in line with previous findings obtained in other countries [3, 7], and the posts analyzed, found through hashtags, had no location restrictions.

It is important that health professionals, including orthodontists, use social media with assertiveness in order to portray greater professional credibility among their followers. The results of the present study highlight important information that can be used by orthodontists to navigate social media with greater professionalism and have a greater impact on the garnering of potential patients.

## Conclusions

Based on the results obtained from the present study, we have concluded the following:

Instagram is a social network widely used by patients, dentistry professionals, and dental students to seek health services, and there is a difference between these groups of the perception of professional credibility portrayed by the orthodontists included in the present study.

Orthodontists and dental students attributed a higher value of professional credibility to images that illustrated technical aspects, in contrast to laypeople and specialists in other areas of dentistry.

Themes related to the orthodontist’s general professional area and before and after images seemed to have a greater influence on the perception of professional credibility, as well as having a higher impact on gaining future patients, as opposed to posts that portrayed their family and social lives.

## Data Availability

The datasets used and/or analyzed during the current study are available from the corresponding author on reasonable request.
